# A Therapeutic Arrangement and Syllabus of Materia Medica

**Published:** 1835-07-01

**Authors:** 


*A Therapeutic Arrangement and Syllabus of Materia Medica.* By James Johnstone, M.D., Fellow of the College of Physicians, and Physician to the General Hospital, Birmingham.—London, 1835. Small 8vo. pp. 84.

This lucid and succinct manual is intended more particularly for the students at the Birmingham School of Medicine, but may prove useful to others; and may even be consulted with advantage by some of the lecturers at the new schools now springing up in all directions. The following is Dr. Johnstone's general arrangement of medicinal substances:

"Class 1. Medicines which act upon the alimentary canal.

"Class 2. Medicines which act upon the glandular system, and upon the secretory and excretory vessels.

"Class 3. Medicines which act upon the heart and arteries.

"Class 4. Medicines which act upon the brain and nervous system.

"Class 5. Medicines which act upon the muscular fibre.

"Class 6. Medicines which act upon the skin and external parts, by application to the surface of the body." (P. 3.)

The following are his arterial stimulants:

"Cantharis.

Abietis resina.

Anisum.

Acorus calamus.

Armoracia.

Balsamum Peruvianum.

Carui semina.

Caryophylli.

Resina flava.

Balsamum Tolutanum.

Benzoinum.

Cajeputi oleum.

Canella.

Cardamomum.

Capsicum.

Rosmarinus.

Cinnamomum.

Copaiba.

Coriandrum.

Cubeba.

Cuminum.

Elemi.

Fœniculum.

Guaiacum.

Lavandula.

Mastiche.

Mentha piperita.

Mezereum.

Myristica.

Olibanum.

Pimenta.

Pix.

Pyrethrum.

Sabina.

Serpentaria.

Styrax.

Sinapis.

Terebinthinæ oleum.

Zingiber.

Æther sulphuricus.

Vinum.

Alcohol.

Chlorinum.

Chloruretum calcis.

Chloruretum sodæ.

Ferrum.

Petroleum.

Piper longum.

Piper nigrum."

(P. 9.)

The nervous stimulants are these:

| | |
|---|---|
| "Ammonia. | Strychnia. |
| Allium porrum. | Opoponax. |
| Allium sativum. | Secale cornutum. |
| Anethum. | Valeriana. |
| Assafœtida. | Toxicodendron. |
| Galbanum. | Æther sulphuricus. |
| Nux vomica. | Sagapenum." (P. 11.) |

In the second part of the work, the medicines "derived from the animal and vegetable kingdoms" are respectively arranged, according to the systems of Cuvier and Jussieu, and the minerals are placed in alphabetical order.

The work concludes with a posological table, containing about four hundred articles. The printer has done his duty as well as the author, and the result has been a very remarkable accuracy, both in names and doses.

